# Effects of Climate Change on the Distribution of *Prosthechea mariae* (Orchidaceae) and within Protected Areas in Mexico

**DOI:** 10.3390/plants13060839

**Published:** 2024-03-14

**Authors:** José Luis Alanís-Méndez, Víctor Soto, Francisco Limón-Salvador

**Affiliations:** 1 Facultad de Ciencias Biológicas y Agropecuarias, Universidad Veracruzana, Región Poza Rica-Tuxpan, Tuxpan 92870, Veracruz, Mexico; lalanis@uv.mx; 2 Centro de Ciencias de la Tierra, Universidad Veracruzana, Xalapa 91090, Veracruz, Mexico; visoto@uv.mx

**Keywords:** climate change, species distribution models, species conservation, endemic plants, *Euchile*

## Abstract

The impact of climate change on the distribution of native species in the Neotropics remains uncertain for most species. *Prosthechea mariae* is an endemic epiphytic orchid in Mexico, categorized as threatened. The objective of this study was to assess the effect of climate change on the natural distribution of *P. mariae* and the capacity of protected areas (PAs) to safeguard optimal environmental conditions for the species in the future. Historical records were obtained from herbaria collections and through field surveys. We utilized climate variables from WorldClim for the baseline scenario and for the 2050 period, using the general circulation models CCSM4 and CNRM-CM5 (RCP 4.5). Three sets of climate data were created for the distribution models, and multiple models were evaluated using the kuenm package. We found that the species is restricted to the eastern region of the country. The projections of future scenarios predict not only a substantial reduction in habitat but also an increase in habitat fragmentation. Ten PAs were found within the current distribution area of the species; in the future, the species could lose between 36% and 48% of its available habitat within these PAs. The results allowed for the identification of locations where climate change will have the most severe effects, and proposals for long-term conservation are addressed.

## 1. Introduction

The increase in greenhouse gas concentrations has been altering the Earth’s climate since the early 20th century. Projections from the Intergovernmental Panel on Climate Change [1] indicate a global average temperature rise of 2–3 °C by the end of this century, with uncertain effects. In Mexico, the consequences of current climate forcing will be experienced to varying degrees across its diverse ecosystems. However, due to the topographical heterogeneity and resulting microclimates, the most pronounced impacts are observed in mountainous regions [2,3,4].

It is worth noting that even minor variations in rainfall patterns and temperature fluctuations can disrupt the ecological balance and pose potential threats to the different species which are dependent on them [5]. Conversely, climatic variability can also benefit certain species, leading to an expansion of their distribution range [6,7,8,9]. Regardless of the intensity and direction of these impacts, both flora and fauna will undergo ecological changes, potentially resulting in shifts in distribution, abundance, range, and ecosystem imbalances due to invasions and public health concerns, among other factors [10,11,12,13].

Several studies have been conducted in global flora research that integrate the spatiotemporal variation of climate elements and the distribution of different species dependent on them. These studies encompass tropical environments [14,15], circumpolar regions [11,16], and mountainous areas [17]. Some of these works are particularly significant as they analyze the climate impact on species with varying threat levels [9,18], especially endemic species [4,19].

In Mexico, protected areas (PAs) have been an effective conservation strategy since 1917 to preserve biodiversity and maintain ecosystem balance, especially for species with varying threat levels [20]. Given the diverse environments in Mexican territory, terrestrial conservation areas are distributed throughout the country, including mountainous regions on the eastern slope, such as the state of Veracruz, which is renowned for its rich and diverse ecosystems [21,22,23].

Species distribution models have proven to be effective predictive tools for spatially representing environmental suitability zones based on physiological requirements at different spatial scales [24,25,26,27]. These models have been refined over the past decade to study evolution, distribution, and conservation [28], particularly in the context of climate change [29,30,31].

The MaxEnt maximum entropy algorithm is the most widely used open-source tool for estimating the potential distribution of species based on the correlation of environmental variables [32,33,34]. In Mexico, MaxEnt has been successfully used to estimate the current and future potential distribution of plants, as well as for conservation purposes [35,36,37,38,39,40].

From a climatic perspective, each species is adapted to specific conditions of temperature, humidity, insolation, and other factors that determine their optimal physiology [41,42,43]. When climate variables surpass these thresholds, species are compelled to persist in suboptimal conditions or migrate to new areas in search of climatic comfort. However, the process of adaptation for plant species, if it occurs, often takes a significant amount of time [44,45,46], thereby exposing species to the risk of extinction during this process [47].

**Figure 1:**
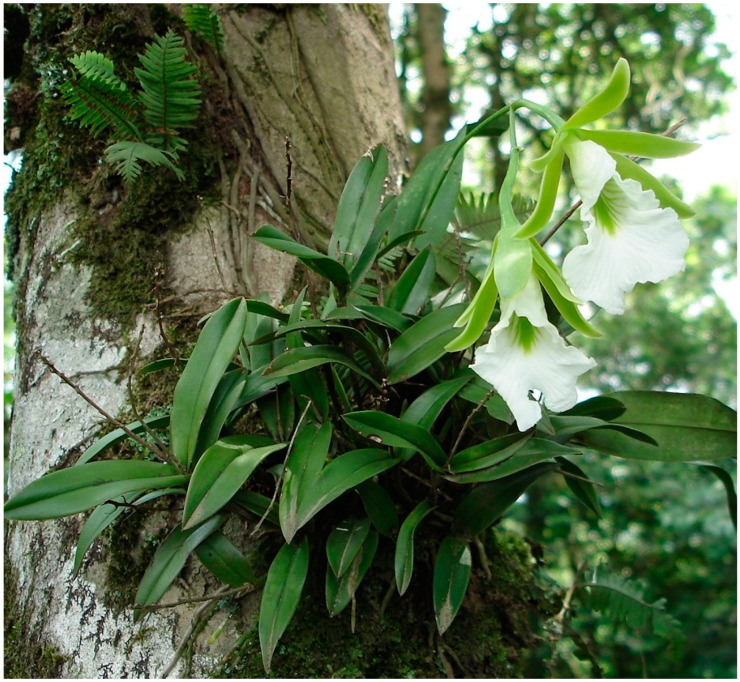
*Prosthechea mariae* (Ames) W. E. Higgins in the protected area, Sierra de Otontepec. Photo by Ivan Rosales Cárdenas.

*Prosthechea mariae* (Ames) W.E.Higgins (Figure 1) also known by the synonyms *Encyclia mariae* (Ames), *Epidendrum mariae* (Ames) or *Euchile mariae* (Ames), is an orchid species endemic to Mexico. This species features diminutive pseudobulbs and white flowers adorned with central green veins. It is an epiphytic species highly valued for its ornamental appeal; consequently, it is subjected to excessive and illegal collection. Moreover, its wild populations have dwindled due to continual habitat destruction.

Despite its endemism and the significant threat it faces, little is known about its distribution. Considering the current climatic variability, this study aims to investigate the potential impacts of climate change on the distribution of the rare and endemic epiphytic orchid *Prosthechea mariae* within Mexico’s protected areas. The study employs the MaxEnt algorithm as the foundation for devising conservation strategies.

## 2. Results

### 2.1. Potential Distribution and Future Scenarios of P. mariae

**Figure 2:**
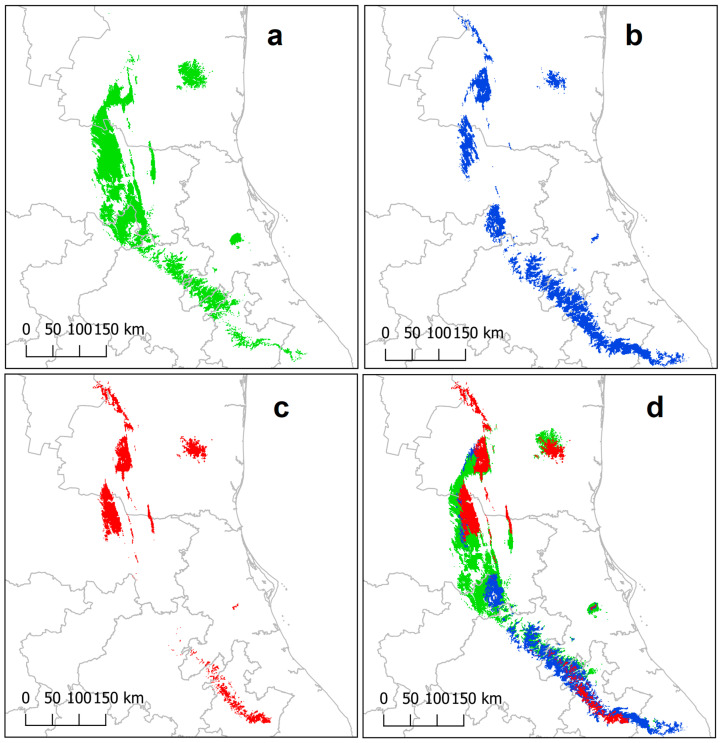
Potential distribution of *P. mariae*: (**a**) green, current distribution model, (**b**) blue, 2050 CNRM-CM5 scenario, (**c**) red, 2050 CCSM4 scenario, and (**d**) ensemble of current and future (2050) potential distribution.

Geographic projections from the models identified an area of 11,434 km^2^ currently suitable for *P. mariae* (Figure 2a). These suitable conditions are primarily found in oak and pine–oak forest vegetation, with small portions also occurring in deciduous lowland jungle and cloud forest. Our models indicate that the species is present in the states of Tamaulipas, San Luis Potosí, Veracruz, Querétaro, and Hidalgo. Additionally, suitable conditions for its distribution were identified in Puebla, where no historical records are available. Based on observation records, the greatest distance between records is 280 linear km.

**Figure 3:**
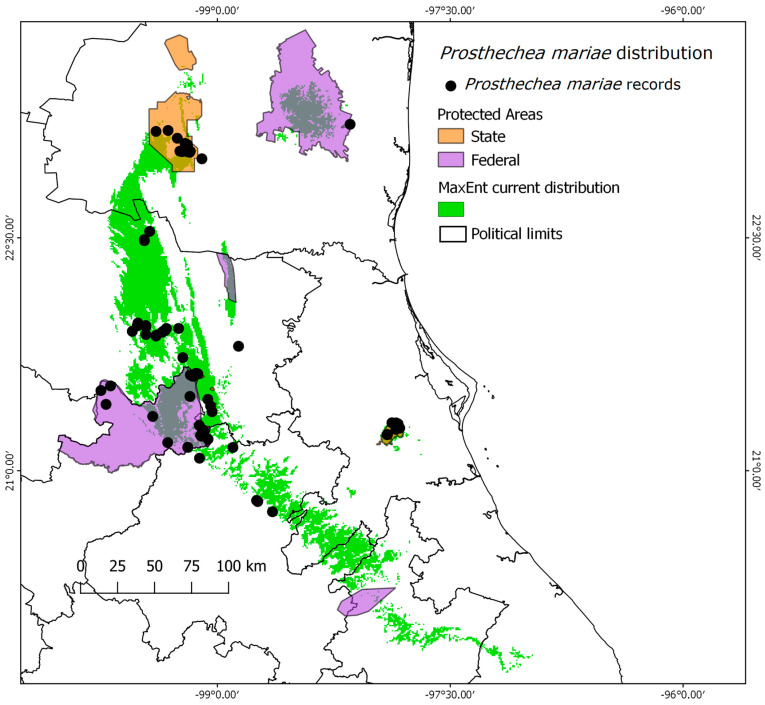
Current potential distribution of *P. mariae* in green color. The protected areas network: State (orange) and Federal (purple) PAs.

**Figure 4:**
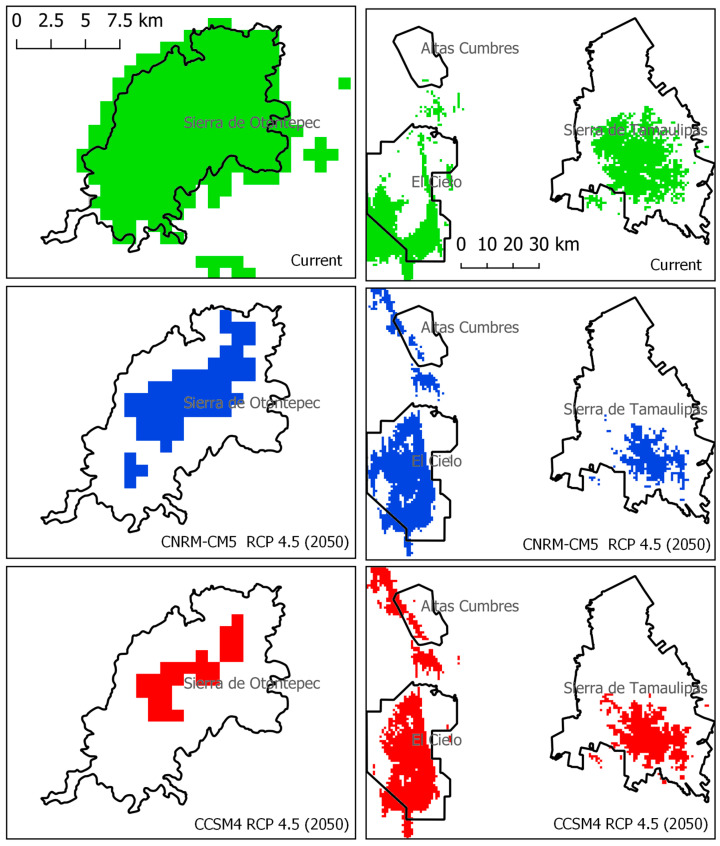
Climate suitability change of *P. mariae* within four protected areas: Altas Cumbres, El Cielo, Sierra de Otontepec, and Sierra de Tamaulipas. Potential current (in green, top) and future (in the middle, CNRM-CM5 model in blue; below, CCSM4 model in red) distributions.

In terms of future climate scenarios, both general circulation models, considering a moderate representative concentration pathway 4.5 (RCP4.5) for the year 2050, consistently predict a reduction in the extent of areas maintaining suitable conditions for *P. mariae* (Figure 2, Figure 3 and Figure 4). The projected suitable area for the species would decrease to 7768 km^2^ (CNRM-CM5) and 4098 km^2^ (CCSM4), representing a loss of optimal conditions for the species’ distribution ranging from 32.06% to 64.16%, respectively.

Furthermore, the projections from both models indicate not only a significant habitat loss but also severe fragmentation, resulting in reduced connectivity. In this scenario, patches of varying sizes with suitable conditions for the species are dispersed throughout the current region of its habitat. These patches are separated by large distances, with at least 90 km between them, as observed in the CCSM4 model (Figure 2c). Both models also predict a latitudinal shift in the species’ distribution, extending towards both the north and south compared to its current range.

### 2.2. Species Protection under the Protected Areas Network

There are ten protected areas (PAs) located within the distribution area of the species, with six being of state domain and four being of federal competence. Combined, these PAs cover an area of 9410 km^2^ (Table 1). Although there are other PAs within the buffer considered in the modeling (area M), they were not included in the results as they do not intersect with the potential distribution of the species.

In the current scenario, these ten PAs collectively safeguard 2718 km^2^ of suitable habitat for the species. This means that the PAs protect 28.89% of the suitable conditions for the species (Table 1). However, under future scenarios, this protected area decreases to 18.25% (CNRM-CM5). In the CCSM4 scenario, the situation is even more concerning, with the protected area decreasing to 15% and resulting in the loss of protection for three PAs (Table 1). These findings indicate that the species could potentially lose between 36.8% and 48.1% of its currently available habitat within the PAs.

## 3. Discussion

The present study is focused on assessing the potential distribution of *Prosthechea mariae* and its conservation within protected areas (PAs) in the face of climate change. Our findings indicate that the species is currently limited to the eastern region of the country, particularly the Huasteca area. However, projected climate change scenarios suggest a significant decrease in the species’ potential distribution, ranging from 32% to 64%. Moreover, the capacity of PAs to safeguard suitable conditions for the species is also expected to decline, protecting only 18.2% to 15% of the habitat by 2050.

### 3.1. Potential Distribution and Future Scenarios of P. mariae

The models identified a potential expansion of the species’ suitable distribution area in the eastern part of the country, particularly in the Huasteca region, encompassing Tamaulipas, San Luis Potosí, Hidalgo, Puebla, and Veracruz [48,49,50]. This finding aligns with historical collection records and information reported by Soto-Arenas [48] regarding the species’ distribution. However, the likelihood of the species being present in the southern distribution, despite favorable environmental conditions indicated by the model, is considered very low. This is primarily due to the absence of records in those parts of Mexico, even though the flower of *P. mariae* is highly distinctive and easily recognizable.

The results demonstrate a reduction in suitable habitat under future climate conditions, with a shift in suitable zones towards the northern with regard to the current distribution, potentially influenced by a less warm climate. Similar to other studies on various biological groups, air warming is expected to drive species migration towards higher latitudes, and the rate of northward migration is positively associated with the degree of air temperature increasing [10,51,52,53].

The findings suggest that climate change poses a significant threat to *P. mariae*, despite the uncertainties inherent in the use of different general circulation models and representative concentration pathways. All model projections predict a drastic reduction in the availability of suitable habitat by 2050 (2041–2060). These projections indicate that areas with suitable climatic conditions for the species will become substantially smaller compared to the current distribution (Figure 2b,c).

Moreover, the projections indicate habitat fragmentation, with climatically suitable conditions for the species being confined to isolated patches of habitat, often separated by distances of 90 km or more (Figure 2b,c). It should be noted that the migration predicted in this study is solely based on the response of *P. mariae* to climate change and does not consider geographical barriers, dispersal capacity, and other factors that could further limit the actual habitat size. Migration capacity plays a crucial role in species’ adaptation to future climate change and is influenced by competition from existing plant communities, anthropogenic habitat fragmentation, and the loss of dispersal agents [54,55].

Despite these unfavorable scenarios, the species may have a chance of survival if it can tolerate and maintain stable populations under climatic conditions different from the current ones [46]. This possibility requires further evaluation through physiological studies, as some species have shown the ability to cope with climate change effects due to inherent phenotypic plasticity in physiological traits [56] or rapid niche shifts [57,58]. However, it is unlikely that niche shifts in *P. mariae* would occur within just five decades, given the limited information available on the genus, which suggests relatively small population sizes and annual generation times [59,60,61].

### 3.2. Analysis of the Environmental Protection of Protected Areas

Climate change is predicted to have global impacts across all environments. However, assessing the effects of climate change on protected areas is a priority for the long-term preservation of species, as these areas are typically established in regions known for their high biodiversity. Previous studies [62,63,64,65] have shown that protected areas can effectively safeguard environmental conditions for certain species, while for others [66,67,68], these conditions may be insufficient. Despite approximately 12% of the world’s terrestrial habitat being covered by protected areas, many of them fail to adequately protect biodiversity and ecological processes [69]. Human activities that modify vegetation within protected areas and their surrounding zones are among the main reasons for this failure [70].

In the case of *P. mariae* conservation, the species becomes more vulnerable due to the anticipated reduction in the proportion of land with suitable climatic conditions within the protected areas network by 2050 (Figure 2, Figure 3 and Figure 4). The models indicate a potential shift in the species’ altitudinal distribution towards higher and cooler conditions, as shown in Figure 4, where the species’ environmental conditions contract spatially and shift latitudinally towards areas with higher humidity and lower temperature.

Even in the current model, the extent of suitable conditions is relatively small, accounting for 28.8% of its total potential distribution. However, the projected situation in the coming decades implies not only a decrease in extent but also the loss of three protected areas that will no longer be able to safeguard the optimal conditions for the species. This highlights the need not only to maintain existing protected areas but also to establish additional conservation areas. It is crucial to preserve habitat heterogeneity within protected areas and their surrounding zones to enable effective management [71]. Clear evidence of forest cover decline in the vicinity of protected areas in tropical regions has been observed in recent decades [72,73,74]. High human population densities and land use practices often isolate protected areas from their surroundings [75].

### 3.3. Ecological Niche Models and Model Evaluation

This study was aimed to reduce uncertainty associated with a single model and explore the future response of *P. mariae* by employing species distribution models with two climate scenarios. Unlike previous studies that relied solely on the default configuration of MaxEnt and a single model, we conducted an extensive exploration of various models, considering criteria that assess the complexity of the generated distribution models [33,76]. The selection of appropriate environmental variables is crucial in species distribution studies as climatic predictors are assumed to be determinants of species distribution.

In our study, we evaluated three distinct combinations, each based on an assumption, and identified the most suitable environmental set for the *P. mariae* data. The dataset 2, composed of a principal component analysis, showed the best model evaluation in our study (see Section 4 and Supplementary File S3). This strategy has proven to be convenient as it allowed us to extract the most information from all available environmental layers, effectively addressing the negative effects of variable collinearity (set 1), as well as objectively discriminating in the variable selection issue (set 3), which is common in studies involving species with heterogeneous responses to environmental variables [77,78].

The distribution of habitat suitability was simulated based on the response of climatic variables. This model does not consider vegetation, land use, among other factors, as variables in the potential distribution of the species [79]. Although it is an approach with limitations, the effects of climate change will directly impact temperature and precipitation. Thus, the obtained results allow for an objective understanding and prediction of the potentially suitable habitat of *P. mariae* under current and future conditions, aiding in making informed decisions for management policies, care, and conservation of this species.

## 4. Materials and Methods

### 4.1. Prosthechea mariae

*Prosthechea mariae* (Figure 1), is an endemic orchid species native to Mexico, with a limited distribution confined to select localities along the country’s eastern slope [80]. According to Soto-Arenas et al. [81], it thrives in a temperate to semi-warm climate with summer rainfall, typically experiencing an average annual temperature ranging from 15 to 22 °C and a significant diurnal temperature variation. In terms of humidity, it requires an annual rainfall exceeding 1000 mm but can withstand dry periods by capitalizing on high relative humidity and condensation in its epiphytic habitats [82].

P. mariae is recognized to inhabit mid-elevation forests (~800–1350 masl), predominantly among oak and liquidambar species. Its presence has been documented in the states of Tamaulipas, San Luis Potosí, Hidalgo, Puebla, and Veracruz [48,49,50]. Its growth occurs between May and November, thriving during the rainy season; flowering takes place in the early summer months, between May and June, with seed dispersal between March and April, during the dry months [83]. Seed maturation necessitates approximately 9 months, while in vitro embryo development spans 4–5 months. Vegetative propagation involves the division of pseudobulbs, typically containing two to three leaves. However, neither of these methods yields a sufficient number of plants to sustain commercialization [84]. Due to its ornamental allure, the species remains a target for uncontrolled and illegal extraction [49], further contributing to its endangered status. In Mexico, this orchid species holds a “threatened species” classification according to NOM-059 SEMARNAT 2010. However, internationally, it lacks protection within the IUCN Red List.

### 4.2. Presence Data of the Species

The occurrence records of *P. mariae* (from year 1956 to 2019) were obtained through physical and digital botanical collections from national herbaria and foreign collections (see Supplementary File S1). Locality records lacking geographic coordinates were georeferenced using Google Earth [85] and converted to decimal degrees using the WGS84 coordinate system. Additionally, data were acquired from the Global Biodiversity Information Facility [86] and the published literature. The historical presence records of the species were enriched through field surveys conducted by our research group and monitoring projects carried out by the group in 2009 and 2019 in the five states previously documented, during the flowering months of the species (May to July).

**Figure 5:**
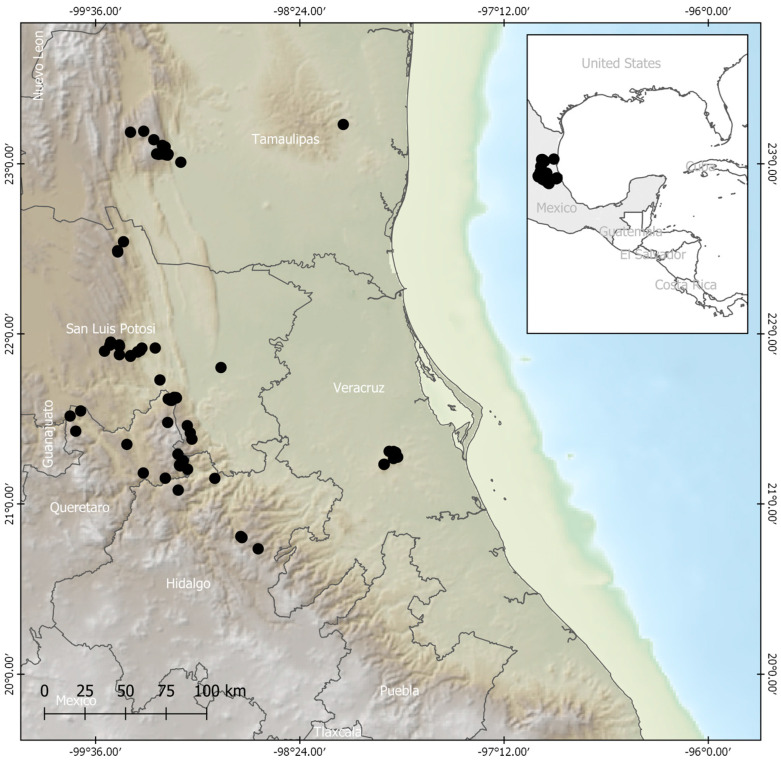
Study area for the distribution of *P. mariae* and its occurrence records.

Only georeferenced data were retained, and the database was refined to remove duplicate entries from herbaria and databases. In total, 116 unique presence records of *P. mariae* were obtained. Prior to modeling, a spatial filtering procedure was applied to the occurrences to mitigate potential spatial autocorrelation effects and prevent overestimation in distribution models [87,88,89]. Considering the limited dispersal capacity of the orchid and the resolution of the environmental information, a filtering distance of 1 km was applied to the original dataset, resulting in 73 records (Figure 5, Supplementary File S2). The selection of records was conducted randomly. The spatial filtering was performed using NTBOX v 0.5.1.0 [90].

### 4.3. Environmental Variables and Future Climate Scenarios

We utilized bioclimatic data from the WorldClim v1.4 platform to ensure comparability in predictions across both spatial and temporal dimensions (current and future). This database comprises 19 climatic variables that encapsulate aspects of precipitation and temperature across the Earth’s surface [91]. Considering multicollinearity, to reduce dimensionality and overfitting in the dataset, the selection of environmental variables was analyzed using three datasets.

In the first dataset, collinearity among the 19 environmental variables was reduced by eliminating variables based on a pairwise Pearson correlation threshold (0.85), resulting in the selection of the following climate layers: BIO1 (annual mean temperature), BIO2 (mean diurnal range), BIO4 (temperature seasonality), BIO12 (annual precipitation), and BIO15 (precipitation seasonality). This procedure was performed using the NTBOX package v 0.5.1.0 [90] within the R programming environment [92]. The second dataset was constructed based on a principal component analysis (PCA) of the 19 layers, using the first five axes that represented 97.23% of the variance in the climatic data. Finally, in the third dataset, all 19 WorldClim layers were left intact. The selection of the best dataset was based on the statistics provided by the R package “kuenm” v1.1.2 [76].

To assess the effects of climate change on the potential distribution of *P. mariae*, the 2050-time period (average for 2041–2060) was selected, and the moderate representative concentration pathway RCP 4.5 was used, avoiding more drastic scenarios (RCP 8.5) or overly optimistic ones (RCP 2.6). Due to the uncertainty associated with future scenarios, climate projections from two general circulation models were used: CCSM4 and CNRM-CM5, which have shown good results for Mexican species [93,94,95,96,97]. Future climate layers were obtained from the same WorldClim v1.4 platform, all with a spatial resolution of 30” (approximately 1 km), and three analogous sets were constructed following the criteria of the present layers.

### 4.4. Ecological Niche Models and Validation

To model the climatic suitability of *P. mariae*, the maximum entropy algorithm was used due to its good performance with presence-only data [25,98,99], implemented in Maxent ver. 3.3.3k [100]. The algorithm estimates the suitability for each pixel given a background sample, under the assumption that the expected value for each feature (i.e., the climatic variables) should be equal to the empirical mean value of the species’ presence points [100,101].

The modeling procedures for the distribution of *P. mariae* took into account the species’ dispersal capacity by considering the BAM framework [102,103] in the background. This framework assumes that the geographic distribution of species is influenced by their biotic relationships (B), abiotic limitations (A), and the potential for dispersal movement (M).

The importance of considering dispersal information in model development has been demonstrated [104]. To avoid unrealistic modeling, the background calibration of the model was restricted to areas assumed to be accessible to the species through dispersal (M area). This area was defined by adding a 200 km buffer around each occurrence point. The selection of this buffering distance took into account the limited dispersal capabilities of both the species and its potential pollinator (which is unknown but possibly a moth), as well as the restricted distribution range of the species according to Soto-Arenas et al. [81]. The resulting extent, known as the M zone, was utilized throughout the study for analyses and future projections [101,104]. It represents a hypothesis about the available zone for future species dispersal, as explained in further detail below.

In the development of ecological niche models, there is currently a focus on maximizing model reproducibility and exploring multiple candidate models [105]. To adhere to this approach, a protocol for calibrating and evaluating the complexity of different candidate models was applied using the R package “kuenm” v1.1.2 [76].

In first instance, the models were executed using 70% of the presence records for model calibration and the remaining 30% for internal evaluation under different combinations of the set of environmental variables. Subsequently, combinations were made by modifying the feature types Linear (L), Quadratic (Q), Product (P), Threshold (T) and Hinge (H), as follows: L, Q, P, T, H, LQ, LP, LT, LH, QP, QT, QH, PT, PH, TH, LQP, LQT, LQH, LPT, LPH, QPT, QPH, QTH, PTH, LQPT, LQPH, LQTH, LPTH, LQPTH, and the values of the regularization multiplier: 0.1, 0.2, 0.3, 0.4, 0.5, 0.6, 0.7, 0.8, 0.9, 1, 2, 3, 4, 5, 6, 8, 10. The changes in each of these parameters and their effect on the construction of the model can be consulted in detail at [101,106]. These combinations were made to evaluate a diverse set of candidate models. The selection of the best models was based on three criteria, first, the statistical significance of the partial ROC test [107], second, the lowest omission rate, and third, the Akaike information criterion (AICc) corrected for small sample sizes [101].

A total of 1479 candidate models were evaluated to assess the climatic suitability for species distribution. These models varied in parameters, including different combinations of regularization multiplier settings (17) and feature class combinations (29), along with three distinct sets of environmental variables (Supplementary File S3). The evaluation of model performance was based on three criteria. Among the total models, 1449 met the criterion of statistical significance (Partial_ROC), and an additional 592 models met the criterion of omission rate (OR). Only one model met the Akaike information criterion corrected for small sample sizes (AICc). Consequently, the best-selected model was M_0.9_F_lqp within Set 2, with the following performance metrics, Mean_AUC_ratio 1.767, Partial_ROC 0, Omission_rate 0.045, AICc 1815.129, and num_parameters 17 (Supplementary File S3).

Using the configuration of the best statistical model, projections were generated for the present time using 100% of the records and 5000 iterations with 10 replicates. These models were then applied to two future climate scenarios (CCSM4 and CNRM-CM5) without extrapolation or clamping (noEC) to avoid artificial projections based on extreme values of ecological variables [34,98,101]. The median values of the replicates were used to summarize the model predictions for each climate scenario. To compare the distribution area between the present and future periods, binary maps were generated using a minimum training presence threshold. All analyses were conducted using version 1.1.5 of the R package “kuenm” v1.1.2 [76].

### 4.5. Analysis of Distribution Patterns

The importance of protected areas (PAs) as spaces capable of preserving optimal bioclimatic conditions for the species was calculated as the proportion of current and future potential distribution areas within PAs systems. For this purpose, layers (shapefile) hosted in CONABIO’s Geoportal, corresponding to state and federal Protected Areas [108] were used and delimited to the region of the study area.

## 5. Conclusions

The current distribution of *P. mariae* is limited to the eastern part of Mexico, primarily within oak and oak–pine forest ecosystems. However, climate change in the medium term (by 2050) will likely lead to habitat reduction and fragmentation, potentially causing the species to migrate to new areas that offer suitable climatic conditions for its survival. This projected impact of climate change on the geographical distribution of suitable areas implies a loss of habitat for *P. mariae*.

The models used to assess future climate scenarios within protected areas indicate that these areas will no longer support the persistence of the species in the coming decades, highlighting the potential failure of some protected areas to fulfill their conservation role for *P. mariae*.

Conserving *P. mariae* poses various challenges, including addressing the impacts of climate change, land use changes, natural and human-induced fires, and the limited knowledge about the species. To ensure the long-term preservation of the species, it is crucial to develop action plans that focus on in situ conservation of the species and its ecosystem, as well as identify potential areas for establishing new protected areas.

## Figures and Tables

**Table 1:** Protected areas that safeguard the environmental conditions of *Prosthechea mariae*.

Protection Type	Name	PAs Decreed Area km^2^	Current Surface Area km^2^ (%)	Projected Surface Area 2050 CNRM-CM5 km^2^ (%)	Projected Surface Area 2050 CCSM4 km^2^ (%)
Federal	Sierra Gorda	3835.67	1142.45 (29.78)	400.21 (10.43)	-
Federal	Sierra de Tamaulipas	3088.88	827.03 (26.77)	324.63 (10.51)	487.26 (15.77)
State	El Cielo	1375.01	451.79 (32.86)	720.49 (52.40)	677.13 (49.25)
Federal	Cuenca Hidrográfica del Rio Necaxa	421.53	10.99 (2.61)	184.69 (43.81)	98.31 (23.32)
State	Altas Cumbres	315.94	2.35 (0.75)	35.84 (11.34)	54.40 (17.22)
Federal	Sierra del Abra Tanchipa	214.84	134.61 (62.65)	3.13 (1.46)	72.71 (33.84)
State	Sierra de Otontepec	151.59	143.41 (94.61)	46.29 (30.53)	21.55 (14.21)
State	La Hoya de las Huahuas	4.07	3.32 (81.61)	-	-
State	El Sótano de Las Golondrinas	2.82	2.82 (100)	2.18 (77.30)	0.08 (2.82)
State	Las Cuevas del Viento y la Fertilidad	0.08	0.08 (100)	-	-
Total		9410.44	2718.85 (28.89)	1717.45 (18.25)	1411.43 (15)

## Data Availability

The data that support the findings of this study are available on request from the corresponding author.

## Supplementary Materials

The following supporting information can be downloaded at: https://www.mdpi.com/article/10.3390/plants13060839/s1, Supplementary File S1. Names of the herbaria and their acronyms consulted to document the presence records of *P. mariae*. Supplementary File S2. 73 unique spatial records used in the analysis of the potential distribution of *P. mariae*. Supplementary File S3. Description of the model calibration and selection process.
