# Supplementary material for: Effects of Climate Change on the Distribution of Prosthechea mariae (Orchidaceae) and within Protected Areas in Mexico
Source: Plants (Basel). 2024 Mar 14;13(6):839. doi: 10.3390/plants13060839 (PMC10974806; doi:10.3390/plants13060839)
Supplement: Supplementary file 1 [file plants-13-00839-s001.zip › Supplementary File S1 Names of the herbaria and their acronyms consulted to document the presence records of P. mariae.pdf]

Effects of climate change on the distribution of *Prosthechea mariae* (Orchidaceae) and within Protected Areas in Mexico.

Supplementary Materials

Supplementary 1. Names of the herbaria and their acronyms consulted to document the presence records of *P. mariae.*

Asociación Mexicana de Orquideología, A. C. (AMO)

Herbario Nacional de México (MEXU)

Herbario del Instituto de Ecología, A.C., México (IE-BAJÍO)

Herbario del Instituto de Ecología, A.C., México (IE-XAL)

New York Botanical Garden (NY)

Herbario Universidad Autónoma Metropolitana (UAMIZ)

Herbario del Real Jardín Botánico (KEW)

Missouri Botanical Garden (MO)

Herbario Isidro Palacios, de la Universidad Autónoma de San Luis Potosí (SLPM)

Herbario Francisco González Medrano de la Universidad Autónoma de Tamaulipas (UAT)

Supplementary 2. 73 unique spatial records used in the analysis of the potential distribution of *P. mariae*.

Supplementary 3. Brief description of the model calibration and selection process.
