# Supplementary material for: Effects of Climate Change on the Distribution of Prosthechea mariae (Orchidaceae) and within Protected Areas in Mexico
Source: Plants (Basel). 2024 Mar 14;13(6):839. doi: 10.3390/plants13060839 (PMC10974806; doi:10.3390/plants13060839)
Supplement: Supplementary file 1 [file plants-13-00839-s001.zip › Supplementary File S3 Description of model calibration and selection process.pdf]

Brief description of the model calibration and selection process

This is the final report of the ku_enm_ceval function implemented in the ku_enm R package.

In all, 1479 candidate models, with parameters reflecting all combinations of 17 regularization multiplier settings, 29 feature class combinations, and 3 distinct sets of environmental variables, have been evaluated. Model peformance was evaluated based on statistical significance (Partial_ROC), omission rates (OR), and the Akaike information criterion corrected for small sample sizes (AICc).

Table S1. Parameters of the candidate models.

	**Parameters**
Regularization multipliers	0.1, 0.2, 0.3, 0.4, 0.5, 0.6, 0.7, 0.8, 0.9, 1, 2, 3, 4, 5, 6, 8, 10
Feature classes	l, q, p, t, h, lq, lp, lt, lh, qp, qt, qh, pt, ph, th, lqp, lqt, lqh, lpt, lph, qpt, qph, qth, pth, lqpt, lqph, lqth, lpth, lqpth
Sets of predictors	Set_1, Set_2, Set_3

The results presented below can be found in the calibration output folder if desired for further analyses.

Model calibration statistics

In the following table is information about how many models met the four selection criteria that this function uses.

Table S2. General statistics of models that met distinct criteria.

Criteria	Number_of_models
All candidate models	1479
Statistically significant models	1449
Models meeting omission rate criteria	592
Models meeting AICc criteria	1
Statistically significant models meeting omission rate criteria	592
Statistically significant models meeting AICc criteria	1
Statistically significant models meeting omission rate and AICc criteria	1

Best models according to user-defined criteria

The following table contains the best models selected according to the user's pre-defined criteria.

Note that if the selection criterion was "OR_AICc", models below the omission rate and among them those with lower AICc values, delta AICc values were recalculated only among models meeting the omission rate criterion (*E*).

Table S3. Performance statistics for the best models selected based on the user's pre-defined criteria.

Model	Mean_AUC_ratio	Partial_ROC	Omission_rate_at_5%	AICc	delta_AICc	W_AICc	num_parameters
M_0.9_F_lqp_Set_2	1.767	0	0.045	1815.129	0	1	17

Model performance plot

The figure below shows the position of the selected models in the distribution of all candidate models in terms of statistical significance, omission rates, and AICc values.

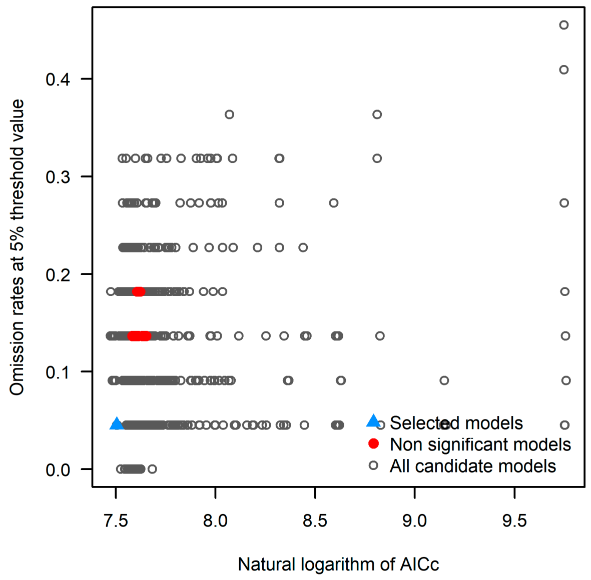

Figure S1. Distribution of all models, non-statistically significant models, and selected models in terms of the user's pre-defined criteria.
